# The Clustering of Smear-Positive Tuberculosis in Dabat, Ethiopia: A Population Based Cross Sectional Study

**DOI:** 10.1371/journal.pone.0065022

**Published:** 2013-05-23

**Authors:** Takele Tadesse, Meaza Demissie, Yemane Berhane, Yigzaw Kebede, Markos Abebe

**Affiliations:** 1 Institute of Public Health, the University of Gondar, Gondar, Ethiopia; 2 Addis Continental Institute of Public Health, Addis Ababa, Ethiopia; 3 Armauer Hansen Research Institute, Addis Ababa, Ethiopia; Wadsworth Center, United States of America

## Abstract

**Background:**

In Ethiopia where tuberculosis epidemic remains high, studies that describe hotspots of the disease are unavailable. This study tried to detect the spatial distribution and clustering of smear-positive tuberculosis cases in Dabat, Ethiopia.

**Methods and Findings:**

A population-based cross sectional study conducted in the Dabat Health and Demographic Surveillance System site from October 2010 to September 2011 identified smear-positive tuberculosis cases. Trained field workers collected demographic and location data from each study participant through house-to-house visits. A spatial scan statistic was used to identify purely spatial and space–time clusters of tuberculosis among permanent residents. Two significant (p<0.001) spatial and space-time clusters were identified in the study district.

**Conclusion:**

Tuberculosis is concentrated in certain geographic locations in Dabat, Ethiopia. This kind of clustering can be common in the country, so the National Tuberculosis Control Program can be more effective by identifying such clusters and targeting interventions.

## Introduction

Tuberculosis (TB) is responsible for a significant morbidity and mortality worldwide, especially in low-income countries. In 2012, the World Health Organization (WHO) estimated that two billion people were infected with TB. Each year, 8.7 million people develop TB and 1.4 million die. Ethiopia is one of the high TB burden countries in the world, with an estimated annual incidence and prevalence of 258 and 237 per 10^5^ population, respectively. The disease mainly affects people who are in the economically productive years of their life (15–59 years), thereby causing considerable social and economic burden on countries [1]. Considering the aggravating factors, such as the human immunodeficiency virus (HIV) co-infection [2] and the emerging drug resistance [3], effective strategies for case detection and cutting transmission are urgently needed.

Tuberculosis control efforts should consider strategies which facilitate reaching the vulnerable population group with less access to health care, and be targeted at high-risk geographical areas and population groups where on-going transmission sustains the epidemic [4].In recent years, new approaches, such as Geographical Information Systems (GIS) and spatial analyses have been used to describe the spatial distribution and clustering of various infectious disease including TB [5]. Different countries of the world, the developed and developing, have used GIS and spatial analysis to study TB disease distribution and have shown a distinct geographical clustering of TB cases, suggesting the likelihood of an ongoing transmission in those areas [4], [6]–[11]).

Although there are many studies on epidemiology of TB in Ethiopia, this is the first attempt at studying the spatial distribution and clustering of smear-positive TB. In a country, where the distribution of risk factors, such as HIV, drug resistance, and social inequities have the greatest heterogeneity, a better understanding of the spatial epidemiology of TB may help to strengthen disease surveillance, to identify risk factors for the spread of the disease, and to plan targeted interventions [5]. Therefore, this study has investigated the spatial distribution and clustering of smear-positive TB in Dabat, Ethiopia.

## Methods

### Setting and population

**Figure 1:**
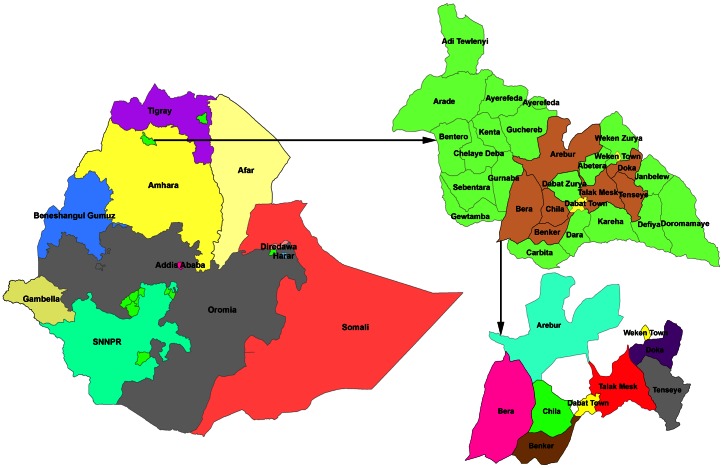
Map of Ethiopia, Dabat District and Dabat HDSSs [Ref.13].

The study was conducted from October 2010 to September 2011 in the Health and Demographic Surveillance System site (HDSSs) located in Dabat district, northern Ethiopia (Figure 1). The Dabat HDSSs consists of three urban and seven rural kebeles (the smallest administrative unit in Ethiopia) with a population of 46,165. There were 9,526 households with an average household size of 4.8[12]. The HDSSs provides a continuous monitoring and updating of events, such as births, deaths, and migration for all household members in the HDSS area. These events are tracked every third months through a longitudinal demographic system by series intervals known as ‘rounds’. The district has 4 health centres and twenty-nine health posts which provide health services for the community. Only two health centers in the study area provided the Directly Observed Treatment Short-course (DOTS) to TB patients at the time of the study. TB treatment involves daily attendance for two months (the intensive phase), followed by a period of four months during which drugs are collected weekly (the continuation phase). Active TB surveillance was introduced in the HDSSs in collaboration with the district health office in October 2010.

### Study design

A cross-sectional study was conducted to detect the spatial distribution and clustering of smear-positive TB in Dabat, Ethiopia, from October 2010 to September 2011.

### Data collection and participants

TB prevalence [13] and incidence [14] studies conducted in Dabat HDSSs preceding the present clustering of smear-positive TB study identified 137 patients aged ≥14 years. Trained field workers collected demographic data (sex, age, place of residence) and clinical data (date of diagnosis, date of registration) from each study participant and DOTS providing health centers using pre-tested and structured questionnaire, respectively. Field workers also collected the geographic location(x-y coordinate) of the residential addresses of all study participants and health facilities in the study area using handheld Global Positioning System (GPS) receivers–Garmin GPS 12 Channel receiver (Garmin Corporation 1998).

### Data management and spatial mapping

Data were entered using Microsoft Access 2003(Microsoft, Redmond, WV, USA). Information on the population (aged ≥14 years and permanent resident) of each study kebele was extracted from the TB Surveillance Project database. To conduct a GIS-based analysis on the spatial distribution of TB, the kebele-level polygon digital map at 1∶100,000 scale was obtained from the Central Statistical Agency of Ethiopia (CSA), on which the kebele-level point layer containing information regarding latitudes and longitudes of central points of each kebele was created. Demographic and clinical data of all study participants were geo-coded and matched to the kebele -level layers of the polygon and point by the administrative code, using the software ArcGIS 9.3(ESRI Inc., Redlands, CA, USA). The output maps were produced using a projected co-ordinate system, UTM Zone 37 North.

### Statistical analysis

Spatial and Space-Time Scan statistics were performed using SaTScanTM v9.1.1 software [15] to test for the presence of statistically significant spatial clusters of TB. The analysis was performed using area-based data aggregated to kebele level. For the purely spatial analysis, case, population, and coordinate data were used as inputs. A Poisson based model was used where the number of events in an area is Poisson distributed according to a known underlying population at risk [16]. The spatial scan statistics works by imposing a circular window on the map and lets the centre of the circle move over the area. At different positions, the window contains different sets of neighbouring areas. The radius of the circular window varies continuously in size from zero up to a maximum so that the window does not include more than 50% of the total population at risk. For this analysis, the maximum spatial cluster size was first set to include up to 50% of the population at risk, which included all smear-positive TB cases diagnosed and permanently lived in the study area between 1 October, 2010 and 30 September, 2011. The spatial cluster size was then set at 25% to test for high excesses and to invesigate the possibility of smaller clusters in the study area. The test of significance of the identified clusters was based on comparing the likelihood ratio test statistics against a null distribution obtained from Monte Carlo Simulation [17]. The number of permutation was set at 999 and the significance level was set at 0.05.

### Ethical considerations

The study protocol was reviewed and approved by the Institutional Review Board (IRB) of the University of Gondar. Government officials at various levels and community leaders were consulted and permission was obtained prior to data collection. The study participants were interviewed after a written informed consent was obtained. Informed written consent regarding eligible subjects below 18 years was obtained from parents or legal guardians. Individual records were coded and accessed only by the research staff.

## Results

**Figure 2:**
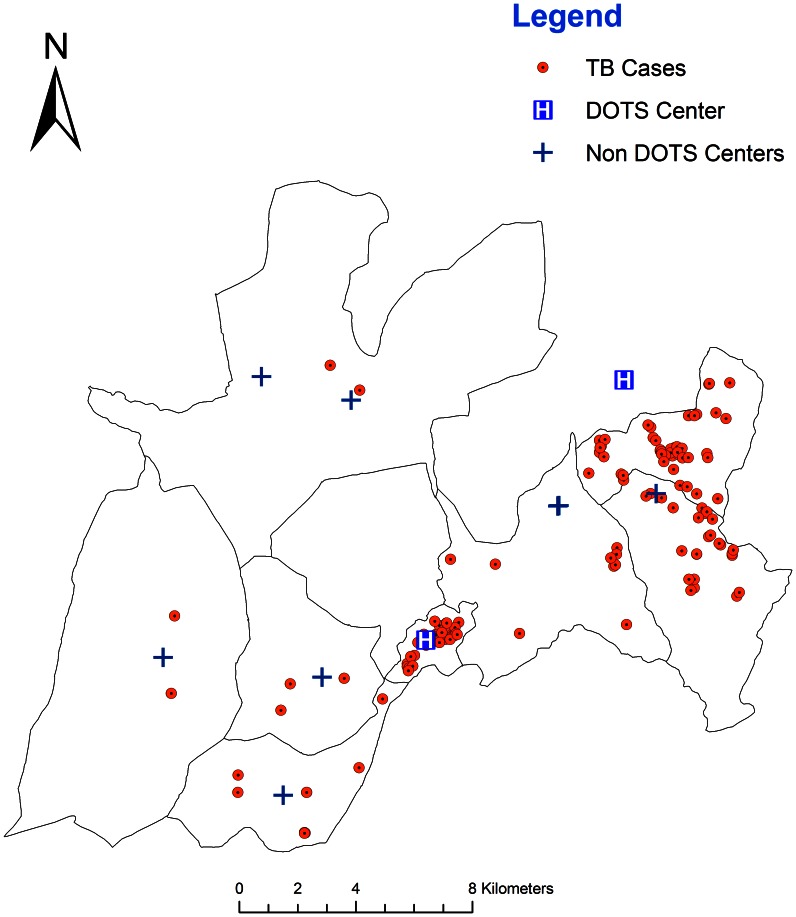
Spatial distribution of TB cases, health centres and health posts in Dabat, Ethiopia, 2012.

Data from a total of 137 smear-positive TB patients were analyzed. The spatial distribution of 137 same-positive TB cases in relation to health facilities in the study area is presented in Figure 2.Even though the distribution does not take the different sizes of the background population into account, it appears that TB cases tend to concentrate in specific geographic areas.

A purely spatial analysis identified a significant clustering of smear-positive TB in two kebeles (neighborhoods), Doka and Tenseye. In order to investigate the possibility of smaller clusters, the same analysis was performed with a maximum spatial cluster size of ≤25% of the total population. The purely spatial analysis identified the most likely significant cluster in the same geographic locations. Furthermore, using a cluster size of ≤50% to scan for areas with low rates of TB showed the presence of three statistically significant low rate of TB at Bera, Arebur and Chilla Kebeles. The number of the observed count, expected count, relative risk, log likelihood, and p-value for each area of excess proportion of smear-positive TB are shown in Table 1.

**Table 1:** Purely spatial clusters with significant higher and lower rates of TB in Dabat, Ethiopia, 2012.

Cluster ID	No. of cases	Expected cases	Relative risk	Log likelihood ratio	P-value
High-rate Most likely cluster	69	26.97	4.12	31.995048	0.001
Secondary cluster	40	34.39	1.23	0.589796	0.855
Low rate Most likely cluster	10	46.70	0.15	27.904473	0.001

### Space-time analysis

**Figure 3:**
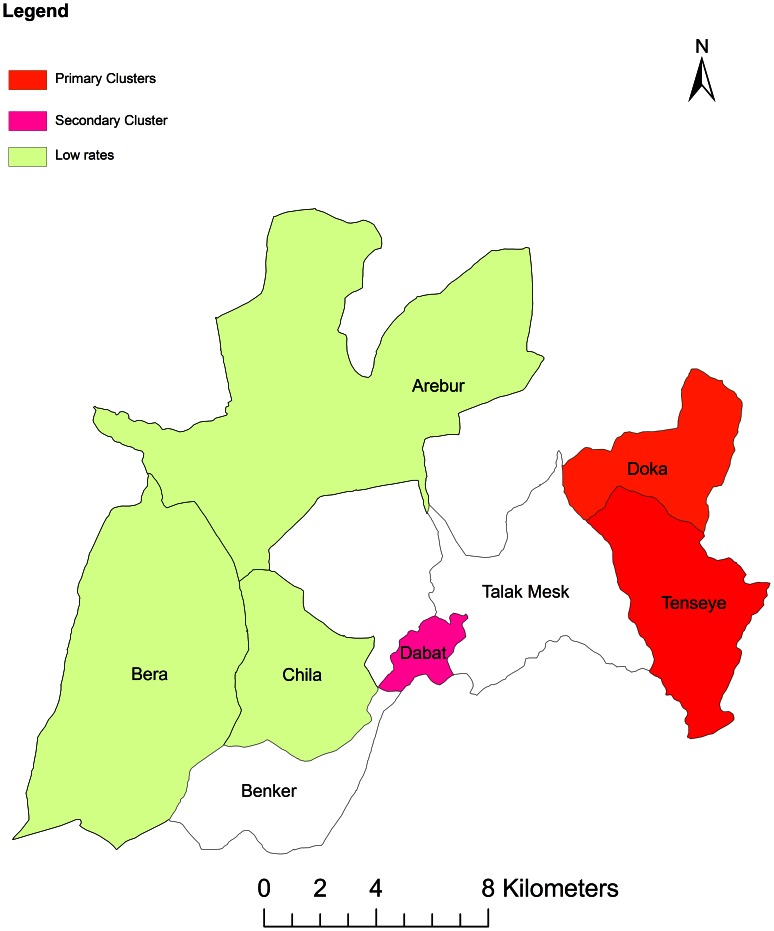
Distributions of high rate and low rate smear-positive TB clusters, in Dabat Ethiopia, 2012.

Using a spatial window that included ≤50% of the population at risk, the most likely statistically significant cluster for high rates of TB was again found to exist in Doka and Tensaye Kebeles between October 2010 and September 2011(RR:10.25, p<0.001), with 59 observed cases and 9.38 expected cases (Table 2). The results of this analysis are presented and highlighted in Figure 3.

**Table 2:** Space-time clusters with significant higher and lower rates of TB in Dabat, Ethiopia 2012.

Cluster ID	No. of cases	Expected cases	Relative risk	Loglikelihood ratio	P-value
High-rate Most likely cluster	59	9.38	10.25	70.017022	0.001
Secondary cluster	9	0.58	16.43	16.471109	0.001

## Discussion

This study investigated the spatial distribution and clustering of smear-positive TB in Dabat, Ethiopia. We mapped TB cases at kebele level to show the spatial distribution of the disease in the study area. The study showed a statistically significant higher rate spatial and space-time clusters in two areas of the district.

In this study, we observed that GIS has a potential to complement the standard monitoring systems in TB control. The mapping of disease patterns using GIS may help to generate hypotheses around transmission (such as hotspots for TB) and provide opportunities for targeted interventions, such as intensified active case-finding. Moreover, a graphical demonstration of the mapping to a wider audience may be a good tool for advocacy and has a role to play in research [18], [19].

This study showed that the spatial distribution of TB in the study area was nonrandom and clustered with a statistical significance (*P*≤0.001) even in a small geographic area, in this case in a kebele. The purely spatial analysis identified two significant spatial clusters for a high incidence of TB. When compared, the purely spatial clusters and the space-time scan statistic, both methods detected similar and significant high-risk clustering in the same geographic area. Consistent results were reported by other studies [4], [6]–[11].The studies showed that TB is distributed in the study area not randomly but in clusters in a spatial pattern.

A disease cluster investigation in space and time may have a role in informing the public health policy [5]. A systematic use of cluster-detection techniques for regular surveillance of TB incidence in Ethiopia may help to develop appropriate strategies, interventions, and tools to improve the quality, coverage, and performance of TB control. This study will also form the background for a series of studies which will explore whether a particular host, organism, and environmental differences may be responsible for clustering through active case detection.

This study analyzed only the statistically significant clusters of the smear-positive TB. Future research is warranted to focus on the effect of various socio-economic and environmental factors on the high incidence of TB in the clustering areas. Disease incidence in clusters may be associated with HIV co-infection [2], unemployment [20], low educational level [21], [22], poor housing quality, crowded living conditions [23], [24], patient factors [25], [26] and alcohol abuse [27].

In conclusion, cases of smear-positive TB clustered geographically in the study setting. This kind of clustering can be common in the country, so the National Tuberculosis Control Program should seek to identify and target such clusters. Factors responsible for higher TB in clusters need to be investigated.
